# Assessing Patient Preferences: Examination of the German Cooper-Norcross Inventory of Preferences

**DOI:** 10.3389/fpsyg.2021.795776

**Published:** 2022-01-14

**Authors:** Peter Eric Heinze, Florian Weck, Franziska Kühne

**Affiliations:** Clinical Psychology and Psychotherapy, University of Potsdam, Potsdam, Germany

**Keywords:** psychotherapy, preference, activity preference, preference assessment, validation study

## Abstract

Despite the positive effects of including patients’ preferences into therapy on psychotherapy outcomes, there are still few thoroughly validated assessment tools at hand. We translated the 18-item Cooper-Norcross Inventory of Preferences (C-NIP) into German and aimed at replicating its factor structure. Further, we investigated the reliability of the questionnaire and its convergence with trait measures. A heterogeneous sample of *N* = 969 participants took part in our online survey. Performing ESEM models, we found acceptable model fit for a four-factor structure similar to the original factor structure. Furthermore, we propose an alternative model following the adjustment of single items. The German C-NIP showed acceptable to good reliability, as well as small correlations with Big-Five personality traits, trait and attachment anxiety, locus of control, and temporal focus. However, we recommend further replication of the factor structure and further validation of the C-NIP.

## Introduction

Psychotherapy is generally effective in the treatment of mental disorders (McAleavey et al., 2019), however, premature treatment termination is still common, with percentages ranging from 20 to 70% (Swift and Greenberg, 2012). Clients often mention dissatisfaction with perceived insufficient therapeutic alliance and therapist’s multicultural competence as a reason for discontinuation (Anderson et al., 2019). Given that treatment dropout rates decrease when patients receive the psychotherapy they consider appropriate (Swift et al., 2018), it is likely that dissatisfied clients were not receiving a therapy that was tailored to their preferences. Even though the APA Task Force on Evidence-Based Practice highlighted that psychotherapeutic preferences should be considered to pursue better therapeutic outcomes (American Psychological Association, 2006), much is still to be desired in the assessment and implementation of patient preferences. Recent instruments to capture preferences are solely available in English, with some questionnaires not being validated thoroughly. Hence, our aim of the study is to provide practitioners and researchers alike with a German tool to capture psychotherapeutic preferences, the German Cooper-Norcross Inventory of Preferences. Furthermore, we validate the questionnaire to investigate the hitherto neglected influence of personality traits and demographics on activity preference choices.

Preferences are defined as anticipatory choices of psychotherapeutic and psychotherapist characteristics that clients wish during psychotherapy (Swift et al., 2011). Preferences are proposed to be multidimensional, dynamic and to operate on different levels of consciousness, i.e., a person might have multiple preferences that are either un-, sub-, or consciously aware and may change over time (Grantham and Gordon, 1986). Currently, preferences are conceptualized into three categories (Swift et al., 2011). First, *treatment preferences* reflect which specific type of intervention patients want. For instance, patients could choose between pharmacological or psychotherapeutic treatment. Second, *activity preferences* capture the clients’ wishes of how they and their psychotherapists should act and behave during psychotherapy (Swift et al., 2018). For example, clients may wish to avoid burdensome topics and want the therapist to lead the psychotherapy. Third, *therapist preferences* indicate which psychotherapist characteristic patients prefer, e.g., regarding gender, race, or personality traits.

Karlsson (2005) summarized particularly relevant methods of preference assessment. In addition to open-ended questions, patients can indicate whether they want treatment by an exemplary therapist that is introduced through vignettes, audiotapes or photos. However, participants might not be aware of their preference or might answer in socially desirable ways based on salient features. Another method described is to present participants with multiple cases and rank-order their ratings on relevant therapy aspects. Methodologically close to this approach is a delay-discounting measure proposed by Swift et al. (2015) allowing for comparisons between two particular characteristics. The more (hypothetical) effectiveness patients sacrifice for any characteristic, the higher their preference. However, external validity is questionable, as preference assessment for characteristics that might not be relevant for patients requires multiple responses to small iterations. Furthermore, in most cases, choosing any preference in an experimental setting does not have an impact on the participants and possible psychotherapy settings. Overall, most methods are well suited for experimental approaches due to their easy applicability and thorough comparability. However, these methods are not always suited to evaluate preferences before starting therapy since the methods mentioned focus on between-group comparisons rather than on individual preferences in consideration of specific treatment circumstances. Therefore, it is necessary to find easily applicable, yet standardized and valid tools to help practitioners and researchers alike to capture preferences of individuals economically.

To this end, different questionnaires are published in English. However, some instruments do not necessarily capture preferences, but therapy-related expectations, and some instruments lack sufficient reliability or validity (for an extended overview: Cooper and Norcross, 2016; Swift et al., 2018).

Therefore, Cooper and Norcross (2016) developed a short and multidimensional measure to be used in clinical practice and research: the Cooper-Norcross Inventory of Preferences (C-NIP). The C-NIP measures clients’ preferences for their therapists’ behavior during psychotherapy or counseling. To avoid response biases in favor of positively keyed items (i.e., people choose high levels of therapist activity regardless of the content), the instrument consists of 18 semantic differential items, i.e., participants choose between two options using a seven-point Likert scale. Whereas positive scores represent stronger preferences toward the left side of the item spectrum and negative scores represent stronger preferences toward the right side, nil scores represent no or the same preference toward both options. Using principal component analysis, the authors identified four subscales: First, *therapist* vs. *client directiveness* expresses whether patients would want the therapist to lead and structure the therapy using psychotherapeutic techniques, or to leave the therapy unstructured and let the patient guide the therapy. This dimension consists of five items that are consistent (Cronbach’s α = 0.84; Cooper and Norcross, 2016). Another five items capture the preference toward e*motional intensity* vs. *reserve*, i.e., the preference for emotion expression and the importance of the therapeutic relationship (α = 0.67). Third, *past* vs. *present orientation* is composed of three items asking the patient whether they want to focus on past or present life events and problems during therapy (α = 0.73). Fourth, *warm support* vs. *focused challenge* consists of five items and captures participants’ preferences toward support and understanding vs. confrontation and challenge (α = 0.60).

As we are not aware of any comparable instruments in German, we thus translated the C-NIP by adhering to established guidelines (Wild et al., 2005) to introduce a tool for German-speaking practitioners and researchers. The guideline subsumes several steps as best practice for translations, i.e., obtaining permissions for translations, independent forward translation as well as backward translation into the source language by a proficient native-speaker, and constant reviews and group discussions after each step. We investigated the factor structure, reliability and construct validity of the C-NIP in a large German sample of laypeople who are the target population of the instrument. Since there are no studies on the relationship between C-NIP preferences and personality so far, we used traits that were identified as predictors of preference choices in previous studies (e.g., Helweg and Gaines, 1977; Petronzi and Masciale, 2015; Anestis et al., 2020). The results could help practitioners to identify patient’s preferences more easily, consider them during therapy, and thus improve therapy outcomes.

However, the association of personality traits with C-NIP preferences is unclear, therefore we used a conservative approach toward hypotheses and expected small effect sizes. We used traits that were identified as particularly relevant for the individualization of psychotherapy, such as adult attachment (Levy et al., 2018), anxiety as an avoidance tendency (Edwards et al., 2019) as well as locus of control (Beutler et al., 2018). From this literature, we infer that anxious participants may prefer reassurance both regarding their relationship with the therapist and concerning the process of psychotherapy (Petronzi and Masciale, 2015). In detail, we first hypothesize small correlations between *trait anxiety*, *attachment anxiety* and *avoidance* with *emotional reserve* and *warm support* (H1). Moreover, as individuals with high *internal locus of control* and *self-efficacy* could expect to be prepared for challenging situations and emotions, we hypothesize small correlations between *internal locus of control* as well as *self-efficacy* with *patient directiveness* and *focused challenge* (H2.1). Furthermore, we expect that *external locus of control* will be associated with *therapist directiveness* and *warm support* (H2.2). Based on findings that Big-Five facets such as *conscientiousness*, *extraversion* and *openness* predict psychotherapy preferences (Ogunfowora and Drapeau, 2008; Petronzi and Masciale, 2015; Anestis et al., 2020), we hypothesize that *conscientiousness* and *extraversion* will be associated with *therapist directiveness* (H3.1) and that *openness* will be associated with *emotional intensity* and *patient directiveness* (H3.2). We assume that preferences for *past* and *present orientation* show small correlations with overall *temporal focus* (H4).

For discriminant validity, due to a lack of prior studies on the validity of the C-NIP and to lower the workload for our participants, we used the same measures (but distinct subscales) as for the investigation of convergent validity. We expected that *temporal focus* does not show significant correlations with C-NIP subscales other than with *past* vs. *present orientation* (H5.1) as well as no other small Big-Five correlations beyond the ones we described above (H5.2). Additionally, based on previous findings that age (Petronzi and Masciale, 2015; Williams et al., 2016), gender (Furnham and Swami, 2008; Ogunfowora and Drapeau, 2008; Liddon et al., 2018), ethnicity (Speight and Vera, 2005; Cabral and Smith, 2011), prior psychotherapeutic experiences (Speight and Vera, 2005; Cooper et al., 2019) and participants’ education (Ogunfowora and Drapeau, 2008; Houle et al., 2013) are significant predictors of preferences, we examined whether preferences differed depending on participant characteristics (e.g., prior psychotherapeutic experience, sociodemographic and personality variables). In detail, we aim to replicate results of Cooper et al. (2019) who found that women have a greater preference for *warm support* than men (H6). Furthermore, mental health professionals showed a greater preference for client directiveness and emotional intensity than did laypersons. Thus, we hypothesize that both participants with prior psychotherapeutic experiences as well as with participants with prior psychological knowledge show greater preferences for *client directiveness* and e*motional intensity* than participants without prior knowledge (H7.1) or experiences (H7.2), respectively.

## Materials and Methods

### Participants

As we expected low overall effect sizes (*d*_*min*_ = 0.20), we aimed for a sample of at least *N* = 779 participants based on a power analysis using GPower 3.1 (two-tailed, statistical power = 0.80; Faul et al., 2009) for correlation analyses, or at least 400 participants per group to perform confirmatory factor analyses (Kyriazos, 2018). Since individuals who are currently in therapy tend to describe their current psychotherapist rather than indicating preferences (Russell et al., 2020) and due to the anticipatory nature of preferences (Grantham and Gordon, 1986), we aimed to recruit a heterogeneous sample irrespective of the participants’ mental status: First, we recruited participants via our department’s participant pool, student mailing lists and social media. *N* = 236 participants were included in this convenience sample. Second, we used the non-commercial SoSci Panel (Leiner, 2016) which is a convenience respondent pool of approximately 80,000 people who consented to be informed about and take part in current surveys and studies without remuneration. After an independent peer review of the study’s approach by the SoSci Panel team, the study link was forwarded to 4,000 panel members, of which we were able to recruit *n* = 733. Overall, three of *n* = 972 participants were excluded from further analyses due to an age younger than 18. Therefore, all subsequent analyses were performed with the total sample of *N* = 969 participants (female: 66.97%; *n* = 649). Mean age was 40.01 years (*SD* = 16.09, range = 18–85). Participants were highly educated (high school diploma or above: 84.5%; *n* = 819), and two thirds had some kind of prior experience with psychotherapy (65.1%; *n* = 627). Only *n* = 24 participants (2.5%) indicated having an ethnic minority background.

Members of the SoSci Panel were significantly older [*t*(756.66) = 22.30, *p* < 0.001], had less prior psychological experiences through jobs or studies [38.9% vs. 64.8%, χ(1) = 47.49, *p* < 0.001], identified themselves more often as religious [44.3% vs. 33.1%, χ(1) = 8.78, *p* < 0.01] and were less politically liberal [*t*(445.33) = 4.41, *p* < 0.001] than non-panel members. Furthermore, panel participants had proportionally fewer females [female: 62.9% vs. 79.7%, χ(2) = 23.10, *p* < 0.001], were less often in training [employed: 44.9% vs. 20.7%, χ(7) = 219.19, *p* < 0.001] and had higher education [master’s degree or equivalent: 37.0% vs. 16.1%, χ(11) = 130.83, *p* < 0.001] than participants of the convenience sample. Whereas members of the SoSci Panel were primarily employed (58.9%; *n* = 432), convenience sample members were primarily undergraduate students (61.9%; *n* = 146).

### Procedure

After obtaining permission to translate the questionnaire by the original author (MC), we translated the C-NIP into German (PH, FK). The initial translation was back translated by an independent English native proficient in psychology (BB). Discrepancies were discussed and consensually resolved within the team of researchers and by including the first author of the original instrument (MC). The study was conducted on the online platform soscisurvey.de (Leiner, 2019). Participants who followed the invitation link gave informed consent. At the end of the study, each participant had the chance to win one out of five 10€-vouchers, and students of the University of Potsdam additionally received course credit. The university’s ethics committee and data protection officer approved the study (no. 13/2020).

### Measures

#### C-NIP

Following the approach on translation and cultural adaptation proposed by Wild et al. (2005), we translated the C-NIP into German (see Supplementary Material). In addition to the semantic differentials, the C-NIP includes 11 open-ended questions on activity and therapist preferences that were translated into German, but not part of the study. For the complete questionnaire and for instructions concerning scoring, please see www.c-nip.net.

#### Relationship Scales Questionnaire

The Relationship Scales Questionnaire (RSQ; Griffin and Bartholomew, 1994; German: Steffanowski et al., 2001) captures attachment styles in adults’ relationships. The questionnaire consists of 30 items using a five point Likert-scale (1 = *not at all like me*, 5 = *very much like me*). Whereas the original authors proposed four subscales, a recent psychometric investigation showed an advantage for two factor models (Zortea et al., 2019). Therefore, we used the two subscales *anxiety* (Cronbach’s α = 0.85) and *avoidance* (α = 0.77).

#### General Self-Efficacy Short Scale (Allgemeine Selbstwirksamkeit Kurzskala)

We used a three item short scale to measure individual general self-efficacy beliefs [Allgemeine Selbstwirksamkeit Kurzskala (AKSU); Beierlein et al., 2017]. Items were rated on a five-point Likert scale (1 = *strongly disagree*, 5 = *strongly agree*). The internal consistency of the ASKU was good (α = 0.89) in the current study.

#### Internal-External-Locus of Control-4 (Internale-Externale-Kontrollüberzeugung-4)

We measured internal and external locus of control using the instrument Internale-Externale-Kontrollüberzeugung-4 (IE-4; Kovaleva, 2012). Participants rated four items using a five-point Likert scale (1 = *strongly disagree*, 5 = *strongly agree*). Since both subscales consist of two items each, we used corrected Spearman-Brown coefficients to investigate reliability (*internal locus of control*: *r* = 0.68; *external locus of control*: *r* = 0.84).

#### State-Trait Anxiety Inventory

Participants rated their trait anxiety on the 20-item State-Trait Anxiety Inventory (STAI; Spielberger et al., 1983; German: Laux et al., 1981) using a four-point Likert scale (1 = *not at all*, 4 = *extremely*). Internal consistency was excellent (α = 0.95).

#### Temporal Focus Scale (Zeitlicher-Fokus-Skala)

Participants rated their cognitive temporal focus on the past or the present on the subscales past focus and present focus of the Zeitlicher-Fokus-Skala (ZFS; Geiger et al., 2018). Its eight items were rated on a seven-point Likert scale (1 = *never*, 7 = *always*). Both 4-item factors *past focus* (α = 0.92) and *present focus* (α = 0.90) showed excellent internal consistencies.

#### Big-Five Inventory (Short Version)

The Big-Five Inventory (BFI-K; Rammstedt and John, 2005) is a 21-item short questionnaire to measure the Big Five personality factors. All items were rated on a five-point Likert scale (1 = *strongly disagree*, 5 = *strongly agree*). Cronbach’s alpha for *Extraversion* (α = 0.85) and *Neuroticism* (α = 0.82) were good, whereas reliabilities for *Agreeableness* (α = 0.65), *Conscientiousness* (α = 0.73) and *Openness* (α = 0.75) were acceptable to questionable.

#### Sociodemographics

Participants indicated their gender (female, male, diverse), education, employment status, religion and ethnicity. Moreover, participants indicated whether they had prior psychotherapeutic experiences or psychological knowledge. Furthermore, we used a ten-point differential with extremes labeled “left” or “right” to measure the political attitude of the participants (Breyer, 2015).

### Data Analyses

To investigate the factor structure, we conducted three analyses: a confirmatory model, a simple exploratory model, and an advanced exploratory model. (1) First, we tested whether our data is suitable for factor analysis as indicated by Kaiser-Meyer-Olkin-criterion (>0.80) and significant Bartlett test. Afterward, we used confirmatory factor analysis (CFA) with four latent factors, no fixed covariances and with weighted least squares (WLSMV) estimator (Sellbom and Tellegen, 2019) to replicate the factor structure reported by Cooper and Norcross (2016). Model fits were determined by the comparative fit index (CFI), the root mean square error of approximation (RMSEA) and the standardized root mean square residual (SRMR). Whereas a CFI of > 0.90 indicates acceptable model fit and CFI > 0.95 indicates a good fit, RMSEA and SRMR values below 0.08 or 0.05 show acceptable or good model fit, respectively (Hu and Bentler, 1999).

(2) As the CFA did not yield acceptable model fit (see section “Results”), we randomly split the data set into two subsamples. First, we extracted the factor structure by performing exploratory factor analysis (EFA) with oblimin rotation using the first subsample. Subsequently, we replicated this model by using CFA on the second subsample.

(3) However, the approach described under section (2) is highly restrictive as it does not allow for cross-loadings of items on different factors, thus constraining the CFA model. Therefore, we performed exploratory structural equation models (ESEM) with WLSMV estimator (Sellbom and Tellegen, 2019). Adding to the first approaches, we did not only implement the factor structure, but also the factor loadings extracted from the initial exploratory factor analysis (EFA) on the first subsample to the ESEM. Again, model fit was assessed using the indices listed above.

For indicating reliability, we computed Cronbach’s alphas for the entire sample. Values above 0.70 indicate acceptable reliability.

For determining construct validity, we used the sum scores according to the best model identified during factor analyses. Convergent and discriminant validity were determined using Pearson’s correlation coefficients. Given the large sample size and power of the analyses, we only interpret correlations exceeding small effect sizes (*r* > 0.10) as meaningful. Group differences (e.g., regarding prior psychotherapeutic experience or sociodemographics) were investigated using independent *t*-tests. All analyses were conducted using R 4.0.2 software (R Core Team, 2020). Data files and scripts are available from the Open Science Framework^1^.

## Results

### Factor Structure

(1) Kaiser-Meyer-Olkin (KMO = 0.84) and a significant Bartlett test showed suitability of our data for factor analyses. The model fits of the first CFA to confirm the factor structure proposed by Cooper and Norcross (2016) did not prove sufficient: RMSEA = 0.090, SRMR = 0.112, CFI = 0.506. When adding fixed covariances derived from the original publication to the model, model fits dropped further due to higher model constraints (RMSEA = 0.099, SRMR = 0.197, CFI = 0.371). Therefore, we conclude that we cannot replicate the factor structure with the German C-NIP translation.

(2) We first performed an exploratory factor analysis with oblimin rotation with a randomly drawn subsample that represented half of the entire sample (*n* = 484). According to PCA, the scree plot and parallel analysis suggested a three-factor-solution. Then, we conducted CFA with a three-latent-factor model with fixed covariances and maximum likelihood estimates on the other half of the data set resulting in insufficient model fit (RMSEA = 0.076, SRMR = 0.123, CFI = 0.582; see Table 1).

**TABLE 1:** Model fits of confirmatory approaches.

				Second subsample (*n* = 485)		
Model		Number of factors	Number of items	*CFI*	*RMSEA*	*SRMR*
PCA + CFA Confirmation						
	Fixed Covariances, Weighted Least Square	3	18	0.582	0.076	0.123
ESEM						
	Free Covariances, Weighted Least Square	4	18	0.922	0.032	0.053
	Free Covariances, Weighted Least Square	4	16	0.959	0.024	0.046
	Free Covariances, Weighted Least Square	3	16	0.869	0.043	0.062

*Second subsample randomly drawn from the entire sample. CFA: confirmatory factor analysis; PCA: principal component analysis; ESEM: exploratory structural equation model.*

(3) We therefore calculated three ESEMs with different specifications, as outlined in Table 1. Replicating the original four-factor structure including all 18 items, model fits were acceptable to good (RMSEA = 0.032, SRMR = 0.053, CFI = 0.922). Therefore, we conclude that the German C-NIP retains a similar factor structure as the English version. The factor loadings for this model are presented in Table 2. However, factor loadings slightly differ from the original English version, i.e., items 6 and 9 load primarily on the first factor and items 10 and 15 have item complexities > 2, i.e., it takes more than two factors to account for each item’s variance. Therefore, we excluded items 10 and 15 to yield better model fits (RMSEA = 0.024, SRMR = 0.046, CFI = 0.959). In this model, items 6 and 9 were reassigned to factor 1, leaving the second factor with only two items (7 and 8) and a more pronounced focus on preferences regarding the therapeutic relationship (see Supplementary Table 2). However, to ease implementation and assessment in clinical practice as well as comparability of studies using different language versions, we recommend using the original factor structure instead of an alternative structure. Therefore, the following results are based on the original factor structure proposed by Cooper and Norcross (2016). For the results on the alternative factor structure, please refer to Supplementary Tables 2,3.

**TABLE 2:** Factor loadings of fitted ESEM-model.

Nr.	Item	TD-CD	EI-ER	PaO-PrO	WS-FC
1	Focus on goals vs. Not focus on goals	**0.62**	0.07	−0.08	0.12
2	Give structure vs. Allow unstructured	**0.65**	0.08	−0.10	0.03
3	Teach skills vs. Not teach skills	**0.89**	−0.04	−0.09	0.00
4	Give homework vs. Not give homework	**0.48**	0.18	−0.03	−0.07
5	Take lead vs. Allow client lead	**0.39**	0.05	0.09	0.02

6	Encourage difficult emotions vs. Not encourage	**0.72**	0.13	0.07	−0.04
7	Talk about relationship vs. Not talk	0.24	**0.63**	−0.02	−0.01
8	Focus on therapy relationship vs. Not focus on therapy relationship	−0.01	**0.71**	0.06	0.05
9	Encourage strong feeling vs. Not encourage	**0.47**	**0.30**	0.13	−0.02
10	Focus on feelings vs. Focus on thoughts	0.22	0.10	**0.37**	0.23

11	Focus on past vs. Focus on present	0.03	−0.01	**0.87**	0.01
12	Reflect childhood vs. Reflect adulthood	0.01	0.04	**0.84**	−0.01
13	Focus on past vs. Focus on future	−0.06	−0.01	**0.90**	0.01

14	Be gentle vs. Be challenging	0.01	−0.06	0.11	**0.48**
15	Supportive vs. Confrontational	**0.50**	−0.12	0.08	**0.41**
16	Not interrupt vs. Interrupt	0.13	−0.03	0.13	**0.49**
17	Not challenge beliefs and views vs. Challenge beliefs and views	−**0.31**	−0.01	−0.05	**0.64**
18	Support behavior unconditionally vs. Challenge behavior	−**0.45**	0.12	−0.01	**0.62**

*Exploratory Factor Analysis with Geomin-Rotation. Bold numbers indicate factor loadings > 0.30. TD-CD: Therapist vs. Client Directiveness; EI-ER: Emotional Intensity vs. Reserve; PaO-PrO: Past vs. Present Orientation; WS-FC: Warm Support vs. Focused Challenge. Horizontal lines separate the factors according to the original English version.*

### Reliability

Cronbach’s alpha for *therapist* vs. *client directiveness* (α = 0.78), *emotional intensity* vs. *reserve* (α = 0.74) and *past* vs. *present orientation* (α = 0.89) were good to acceptable, whereas the reliability of *warm support* vs. *focused challenge* (α = 0.65) was questionable.

### Convergent Validity

Descriptive statistics and correlations are presented in Table 3. Overall, correlation coefficients were small, with eight correlations exceeding the limit for small effect sizes of *r* > 0.10. As expected, *attachment avoidance* was associated with *emotional reserve* (H1), and *external locus of control* correlated with *warm support* (H2.2). Furthermore, temporal focus on past or present was associated with *past or present orientation*, respectively (H4). The significant correlations between *attachment anxiety* and *avoidance* with *warm support*, *trait anxiety* with *emotional reserve* and *warm support* (H1) as well as *conscientiousness* with *therapist directiveness* (H3.1) and *openness* with *emotional intensity* (H3.2) were according to our hypotheses, but failed to exceed the threshold of relevant effect sizes (*r* > 0.10). Contrary to our hypotheses, there were no relevant associations between *attachment anxiety* and *emotional reserve* (H1), *internal locus of control* and *focused challenge* (H2.1), *extraversion* and *therapist directiveness* (H3.1) as well as *openness* and *patient directiveness* (H3.2).

**TABLE 3:** Descriptive and correlations with C-NIP scale sums.

Scale		*M*	SD	α	*r(TD-CD)*	*r(EI-ER)*	*r(PaO-PrO)*	*r(WS-FC)*
C-NIP								
	Therapist vs. Client Directiveness	6.85	5.36	0.78	1			
	Emotional Intensity vs. Reserve	6.07	5.00	0.74	0.53***	1		
	Past vs. Present Orientation	−0.53	4.39	0.89	0.13***	0.35***	1	
	Warm Support vs. Focused Challenge	−1.38	5.05	0.65	0.02	0.14***	0.38***	1
Relationship Scales Questionnaire								
	Anxiety	2.43	0.91	0.85	−0.08**	−0.02	0.14***	0.08**
	Avoidance	2.37	0.86	0.77	−0.09**	−0.10**	0.06	0.08*
General Self-Efficacy								
	Overall	3.97	0.72	0.89	0.06	0.04	−0.07*	−0.06
Locus of Control								
	Internal	3.93	0.77	0.68	0.15***	0.09**	−0.04	−0.02
	External	2.35	0.84	0.58	−0.07*	−0.07*	0.06	0.10**
Trait Anxiety								
	Overall	2.08	0.62	0.95	−0.09**	−0.09**	0.08*	0.09**
Temporal Focus								
	Past	3.75	1.15	0.92	−0.03	−0.02	0.15***	0.04
	Present	4.92	1.08	0.90	0.06	0.06	−0.11***	−0.07*
Big Five								
	Extraversion	3.33	0.95	0.85	0.04	0.12***	0.02	−0.08*
	Agreeableness	3.16	0.78	0.65	0.02	0.10**	−0.02	0.00
	Conscientiousness	3.72	0.74	0.73	0.09**	0.04	−0.01	−0.02
	Neuroticism	3.09	0.98	0.82	−0.09**	−0.07*	0.08*	0.09**
	Openness	4.05	0.69	0.75	−0.02	0.07*	−0.01	−0.06

*Correlations show Pearson’s correlation coefficients. Negative correlations resemble increasing preference toward the right anchor of each C-NIP’s scales. Bold correlation coefficients mark (at least) small effect sizes (r > 0.10). TD-CD, Therapist vs. Client Directiveness; EI-ER, Emotional Intensity vs. Reserve; PaO-PrO, Past vs. Present Orientation; WS-FC, Warm Support vs. Focused Challenge.*

*^1^Spearman-Brown Coefficient due to 2 item scale.*

*p < 0.05; **p < 0.01; and ***p < 0.001.

### Discriminant Validity

As hypothesized, *temporal focus* did not correlate with any scale other than *past* vs. *present orientation*, except for an association between *past focus* and *attachment intensity* (H5.1). Contrary to our hypothesis, *extraversion* and *agreeableness* were correlated with *emotional intensity* (H5.2).

### Group Differences Regarding Individual Variables

As expected, women preferred less *focused challenge* [*M* = −1.11 vs. −1.96; *t*(675.04) = −2.58, *p* < 0.05, *d* = 0.17] than men (H6). Participants with previous psychological knowledge preferred more *emotional intensity* than participants without previous psychological knowledge [H7.1; 6.59 vs. 5.67; *t*(896.65) = −2.84, *p* < 0.001, *d* = 0.19]. The same pattern emerged for participants with prior psychotherapeutic experiences preferring more *emotional intensity* than participants with no experiences [H7.2; 6.38 vs. 5.60; *t*(662.30) = −2.30, *p* < 0.05, *d* = 0.16].

On an exploratory level, there emerged small, significant correlations between older age and *emotional intensity* (*r* = 0.12, *p* < 0.001). There is also a small, significant association between higher education and preferences toward *present orientation* (τ = −0.11, *p* < 0.001). There were no significant or meaningful associations between the C-NIP factors and religiosity, ethnicity and political attitude.

## Discussion

We translated the Cooper-Norcross Inventory of Preferences (Cooper and Norcross, 2016) into German and aimed for a replication of the factor structure and an investigation of the nomological network of the questionnaire using a large, heterogeneous sample. In addition to translations into other languages such as Portuguese, French and Turkish, our study represents one of the first independent and elaborate investigations of the C-NIP of this magnitude. We found that a CFA conducted in an independent sample did not support the original factor structure. However, ESEM models indicated good to acceptable model fit indices for a similar 18-item, 4-factor structure. Furthermore, we identified an improved alternative 4-factor model in which items 10 and 15 were excluded, and items 6 and 9 were reassigned to a different factor.

Just as the Portuguese, French and Turkish C-NIP translations, we were not able to replicate the original factor structure (Malosso, 2019; Volders, 2021; Özer and Yalçın, 2021). As one explanation for divergent results, the authors of the original C-NIP performed a single PCA to extract suitable items out of a 40-item pool. Thus, it is likely that factor loadings will change if another PCA is performed using the 18-item version. Second, Cooper and Norcross recruited a sample mainly consisting of psychotherapy experts, whereas we included laypersons, as they are defined as the target population of the C-NIP. However, our sample was quite similar to the original one since two-thirds of our sample reported having prior experiences with psychotherapy. Third, there might be cultural differences, even though our group followed the approach on (back) translation and cultural adaptation by Wild et al. (2005) which should have contributed to comparability. Still, items 10 and 15 showed significant cross-loadings and high item complexity. Both items were also difficult to integrate in the factor structures of other translations. For example, item 10 of the French translation primarily loaded on the scale *therapist* vs. *client directiveness* instead of the factor *emotional intensity* vs. *reserveness* (Volders, 2021). In the Portuguese version, item 15 was excluded as it did not contribute significantly to the factor *warm support* vs. *focused challenge* (Malosso, 2019). In line with these studies, we assume different reasons for the cross-loadings: Whereas all items describe a dichotomy of preferring a certain behavior or not, item 10 (focus on emotions vs. focus on thoughts) differs from this pattern. The content of item 15 (be supportive vs. be confrontational) could be mistaken as supportiveness through directiveness, i.e., rather than being emotionally supportive, a therapist could support the patient by structuring the therapy or by giving homework. Above, we argue that two items (6, 9) previously belonging to the factor *emotional intensity* vs. *reserve* could be reassigned to the first factor *therapist* vs. *client directiveness*. Content wise, both items focus on the preference whether the therapist should encourage the patient to go into emotions or feelings, respectively. In our view, both items more closely match the facet of directiveness. Therefore, we are left with two items of the former *emotional intensity* vs. *reserve* facet that both focus on how therapists are supposed to manage the therapeutic relationship. This factor could indicate whether the participants want the therapist to focus on the therapeutic alliance. Due to its consistently found positive effects on therapy outcomes (Flückiger et al., 2018), it seems reasonable to have a distinct factor focusing on this aspect of psychotherapy.

We found several expected correlations between the C-NIP factors and trait variables. For example, *temporal focus* on past or present was associated with preferences toward *past or present orientation*, respectively (H4). As expected, *attachment avoidance* was related to *emotional reserve* (H1), and *external locus of control* was correlated with *warm support* (H2.2). However, eight correlations barely exceeded the threshold of small effect sizes (*r* > 0.10), and most significant correlations (*n* = 20) even failed to cross the threshold. Therefore, the results suggest that personality may play a significant, yet minor role concerning preference choices. Moreover, due to a more detailed and facet-oriented approach, a few results are contrary to our hypotheses and to previous findings on treatment preferences. For example, *extraversion* was associated with *emotional intensity* that could be ascribed to represent a psychodynamic rather than a CBT approach (H5.2; Petronzi and Masciale, 2015). However, this result does not necessarily counter the results of previous studies, but rather shows that it is not sufficient to ask for preferences toward a specific treatment approach. Instead, future studies on treatment preferences should also implement therapist activity preferences, i.e., preference toward specific behavior of the therapist.

According to our study, participants high in attachment avoidance, and, to a smaller degree, attachment and trait anxiety, preferred a gentle and supportive approach in psychotherapy. However, past studies on anxiety disorders found that psychotherapy is often preferred over pharmacological treatment during which no confrontation with the anxiety-inducing stimuli is necessary (Mohlman, 2012; Arch, 2014). We assume that, at this point, laypersons and patients might be aware that psychotherapy including exposition interventions is the most effective treatment of anxiety (Mayo-Wilson et al., 2014). As some patients prefer to be treated gently in advance, it is important to measure patients’ preferences and concerns with standardized methods such as the C-NIP in order to adjust the therapeutic process to an equilibrium between effective, evidence-based treatments and the accommodation of patients’ preferences.

Overall, the effect sizes were too small to clearly determine construct validity of the instrument. However, to our knowledge, our study presents the first comparison of the C-NIP with diverse personality questionnaires. Relatively stable trait measures such as trait anxiety, adult attachment and locus of control might fail to capture the dynamic nature of preferences (Grantham and Gordon, 1986). As personality measures seemed inadequate to determine construct validity, further investigations might rather use less stable constructs such as expectations, current mood or well-being.

### Limitations and Future Directions

Recruiting a large heterogeneous sample of *N* = 969 laypeople, the sample size goes along with well-powered analyses. In order to avoid false positive results, we limited our interpretation to correlations exceeding small effect sizes (*r* > 0.10). Overall, participants were highly educated with 84.5 per cent of our sample holding at least a high-school diploma. Therefore, our results and interpretations are limited and need to be replicated with different samples including participants with more heterogeneous educational backgrounds. Moreover, although two thirds of our sample indicated that they had some kind of prior psychotherapeutic experiences, we did not recruit a patient sample. Previous studies showed that patients’ preferences are similar to their actual psychotherapists (Russell et al., 2020), thus we included laypersons perspectives so that biases due to current symptoms and ongoing psychotherapeutic treatments are less probable. Like in the original publication, the C-NIP factor *warm support* vs. *focused challenge* showed questionable reliability of <0.70. Furthermore, overall means of each factor are significantly different from zero. As Cooper and Norcross (2016) point out, this result merely represents a preference toward *therapist directiveness*, *emotional intensity*, *present orientation* and *focused challenge* in our sample. Furthermore, the questionnaire might not capture every aspect that is relevant for a patient. In practice, if patients did not think about their preference yet, the C-NIP might act as a facilitator for reflection. Furthermore, it might help therapist to explain their approach, to individualize therapy or to clear out misconceptions.

Due to the above-mentioned issues regarding validity and factor structure, we strongly recommend further replication studies by independent researchers. Still, the implementation of the C-NIP into clinical practice might prove useful in order to investigate its clinical utility and its impact on variables such as therapeutic alliance or treatment termination. We propose implementing the C-NIP after making a first appointment and before the first therapy session to minimize potential biases. A longitudinal study of patient preferences during the course of psychotherapy could shed light on preferences’ variability as an important aspect of managing and guiding the therapy.

### Conclusion

Overall, the reliability, validity and factor structure of the German Cooper-Norcross Inventory show promising results, yet there is room for improvement. To date, research lacks replication of the original factor structure as well as evidence for the instrument’s validity and usefulness for research purposes. However, first small associations with personality traits hint at its usefulness. Thus, the instrument needs further independent investigations of its psychometric properties as well as on its practical utility in different clinical samples.

## Data Availability Statement

The datasets presented in this study can be found in online repositories. The names of the repository/repositories and accession number(s) can be found below: osf.io/n6xbq/.

## Ethics Statement

The studies involving human participants were reviewed and approved by Ethics Committee and Data Protection Officer of the University of Potsdam (13/2020). The patients/participants provided their written informed consent to participate in this study.

## Author Contributions

PH and FK performed the material preparation, data collection, and analysis. PH wrote the first draft of the manuscript. All authors contributed to the study conception and design, commented on previous versions of the manuscript, and read and approved the final manuscript.

## Conflict of Interest

The authors declare that the research was conducted in the absence of any commercial or financial relationships that could be construed as a potential conflict of interest.

## Publisher’s Note

All claims expressed in this article are solely those of the authors and do not necessarily represent those of their affiliated organizations, or those of the publisher, the editors and the reviewers. Any product that may be evaluated in this article, or claim that may be made by its manufacturer, is not guaranteed or endorsed by the publisher.

## Footnotes

1
https://osf.io/n6xbq

## References

[B1] American Psychological Association (2006). Evidence-based practice in psychology. *Am. Psychol.* 61:271. 10.1037/0003-066X.61.4.271 16719673

[B2] Anderson K. N. Bautista C. L. Hope D. A. (2019). Therapeutic alliance, cultural competence and minority status in premature termination of psychotherapy. *Am. J. Orthopsychiatry* 89 104–114. 10.1037/ort0000342 30010364

[B3] Anestis J. C. Rodriguez T. R. Preston O. C. Harrop T. M. Arnau R. C. Finn J. A. (2020). Personality assessment and psychotherapy preferences: Congruence between client personality and therapist personality preferences. *J. Personal. Assess.* 2020 1–11. 10.1080/00223891.2020.1757459 32364800

[B4] Arch J. J. (2014). Cognitive behavioral therapy and pharmacotherapy for anxiety: Treatment preferences and credibility among pregnant and non-pregnant women. *Behav. Res. Therapy* 52 53–60. 10.1016/j.brat.2013.11.003 24326075

[B5] Beierlein C. Kemper C. J. Kovaleva A. Rammstedt B. (2017). Short scale for measuring general self-efficacy beliefs (ASKU). *Methods Data Anal.* 7:28. 10.12758/mda.2013.014

[B6] Beutler L. E. Kimpara S. Edwards C. J. Miller K. D. (2018). Fitting psychotherapy to patient coping style: A meta-analysis. *J. Clin. Psychol.* 74 1980–1995. 10.1002/jclp.22684 30198566

[B7] Breyer B. (2015). *Left-Right self-placement (ALLBUS). Zusammenstellung Sozialwissenschaftlicher Items Und Skalen.* Mannheim: GESIS – Leibniz-Institut, 10.6102/zis83

[B8] Cabral R. R. Smith T. B. (2011). Racial/ethnic matching of clients and therapists in mental health services: A meta-analytic review of preferences, perceptions, and outcomes. *J. Counsel. Psychol.* 58 537–554. 10.1037/a0025266 21875181

[B9] Cooper M. Norcross J. C. (2016). A brief, multidimensional measure of clients’ therapy preferences: The Cooper-Norcross Inventory of Preferences (C-NIP). *Int. J. Clin. Health Psychol.* 16 87–98. 10.1016/j.ijchp.2015.08.003 30487853 PMC6225020

[B10] Cooper M. Norcross J. C. Raymond-Barker B. Hogan T. P. (2019). Psychotherapy preferences of laypersons and mental health professionals: Whose therapy is it? *Psychotherapy* 56 205–216. 10.1037/pst0000226 31094538

[B11] Edwards C. J. Beutler L. E. Someah K. (2019). “Reactance level,” in *Psychotherapy Relationships that Work: Volume 2: Evidence-Based Therapist Responsiveness*, eds Norcross J. C. Wampold B. E. (Oxford: Oxford University Press), 188–211.

[B12] Faul F. Erdfelder E. Buchner A. Lang A.-G. (2009). Statistical power analyses using G*Power 3.1: Tests for correlation and regression analyses. *Behav. Res. Methods* 41 1149–1160. 10.3758/BRM.41.4.1149 19897823

[B13] Flückiger C. Del, Re A. C. Wampold B. E. Horvath A. O. (2018). The alliance in adult psychotherapy: A meta-analytic synthesis. *Psychotherapy* 55:316. 10.1037/pst0000172 29792475

[B14] Furnham A. Swami V. (2008). Patient preferences for psychological counsellors: Evidence of a similarity effect. *Counsel. Psychol. Quart.* 21 361–370. 10.1080/09515070802602146

[B15] Geiger S. M. Domenech F. van der Meer E. (2018). *Eine Skala zur Messung des zeitlichen Fokus (ZFS). Deutsche Version der Temporal Focus Scale. Zusammenstellung Sozialwissenschaftlicher Items Und Skalen.* Mannheim: GESIS – Leibniz-Institut, 10.6102/zis259

[B16] Grantham R. J. Gordon M. E. (1986). The nature of preference. *J. Counsel. Dev.* 64:396. 10.1002/j.1556-6676.1986.tb01146.x

[B17] Griffin D. W. Bartholomew K. (1994). “The metaphysics of measurement: The case of adult attachment,” in *Attachment processes in adulthood*, Vol. 5 eds Bartholomew K. Perlman D. (London: Jessica Kingsley Publishers), 17–52.

[B18] Helweg G. C. Gaines L. S. (1977). Subject characteristics and preferences for different approaches to psychotherapy: A multivariate study. *J. Consult. Clin. Psychol.* 45 963–964. 10.1037/0022-006X.45.5.963 903462

[B19] Houle J. Villaggi B. Beaulieu M.-D. Lespérance F. Rondeau G. Lambert J. (2013). Treatment preferences in patients with first episode depression. *J. Affect. Disord.* 147 94–100. 10.1016/j.jad.2012.10.016 23167975

[B20] Hu L. Bentler P. M. (1999). Cutoff criteria for fit indexes in covariance structure analysis: Conventional criteria versus new alternatives. *Struct. Equat. Model. Multidiscipl. J.* 6 1–55. 10.1080/10705519909540118

[B21] Karlsson R. (2005). Ethnic matching between therapist and patient in psychotherapy: An overview of findings, together with methodological and conceptual issues. *Cult. Divers. Ethnic Minor. Psychol.* 11 113–129. 10.1037/1099-9809.11.2.113 15884983

[B22] Kovaleva A. (2012). *The IE-4: Construction and validation of a short scale for the assessment of locus of control.* Mannheim: GESIS – Leibniz-Institut.

[B23] Kyriazos T. A. (2018). Applied Psychometrics: Sample size and sample power considerations in factor analysis (EFA, CFA) and SEM in general. *Psychology* 09:2207. 10.4236/psych.2018.98126

[B24] Laux L. Glanzmann P. Schaffner P. Spielberger C. D. (1981). *Das State-Trait-Angstinventar (Testmappe mit Handanweisung, Fragebogen STAI-G Form X1 und Fragebogen STAI-G Form X2).* Weinheim: Beltz.

[B25] Leiner D. J. (2016). Our research’s breadth lives on convenience samples: A case study of the online respondent pool “SoSci Panel.”. *Stud. Commun. Med.* 5 367–396. 10.5771/2192-4007-2016-4-367 20113525

[B26] Leiner D. J. (2019). *SoSci Survey (3.1.06).* Available online at: https://www.soscisurvey.de (accessed April 20, 2020).

[B27] Levy K. N. Kivity Y. Johnson B. N. Gooch C. V. (2018). Adult attachment as a predictor and moderator of psychotherapy outcome: A meta-analysis. *J. Clin. Psychol.* 74 1996–2013. 10.1002/jclp.22685 30238450

[B28] Liddon L. Kingerlee R. Barry J. A. (2018). Gender differences in preferences for psychological treatment, coping strategies, and triggers to help-seeking. *Br. J. Clin. Psychol.* 57 42–58. 10.1111/bjc.12147 28691375

[B29] Malosso M. S. (2019). *Adaptação transcultural para o português e validação de duas ferramentas de avaliação das preferências do cliente em psicoterapia: C-NIP e PEX.P.* Lisboa: ISPA-Instituto Universitario.

[B30] Mayo-Wilson E. Dias S. Mavranezouli I. Kew K. Clark D. M. Ades A. E. (2014). Psychological and pharmacological interventions for social anxiety disorder in adults: A systematic review and network meta-analysis. *Lancet Psychiatry* 1 368–376. 10.1016/S2215-0366(14)70329-3 26361000 PMC4287862

[B31] McAleavey A. A. Youn S. J. Xiao H. Castonguay L. G. Hayes J. A. Locke B. D. (2019). Effectiveness of routine psychotherapy: Method matters. *Psychother. Res.* 29 139–156. 10.1080/10503307.2017.1395921 29096584

[B32] Mohlman J. (2012). A community based survey of older adults’ preferences for treatment of anxiety. *Psychol. Aging* 27 1182–1190. 10.1037/a0023126 21463061

[B33] Ogunfowora B. Drapeau M. (2008). A study of the relationship between personality traits and theoretical orientation preferences. *Counsel. Psychother. Res.* 8 151–159. 10.1080/14733140802193218

[B34] Özer Ö Yalçın I. (2021). Cooper-Norcross Tercih Envanteri Türkçe Uyarlaması: Bir Geçerlik ve Güvenirlik Çalışması. *Anadolu J. Educat. Sci. Int.* 11 26–44. 10.18039/ajesi.790673

[B35] Petronzi G. J. Masciale J. N. (2015). Using personality traits and attachment styles to predict people’s preference of psychotherapeutic orientation. *Counsel. Psychother. Res.* 15 298–308. 10.1002/capr.12036

[B36] R Core Team (2020). *R: A language and environment for statistical computing [Computer software].* Vienna: R Foundation for Statistical Computing.

[B37] Rammstedt B. John O. P. (2005). Kurzversion des Big Five Inventory (BFI-K): Entwicklung und Validierung eines ökonomischen Inventars zur Erfassung der fünf Faktoren der Persönlichkeit. *Diagnostica* 51 195–206. 10.1026/0012-1924.51.4.195

[B38] Russell K. A. Swift J. K. Penix E. A. Whipple J. L. (2020). Client preferences for the personality characteristics of an ideal therapist. *Counsel. Psychol. Quart.* 2020 1–17. 10.1080/09515070.2020.1733492

[B39] Sellbom M. Tellegen A. (2019). Factor analysis in psychological assessment research: Common pitfalls and recommendations. *Psychol. Assess.* 31 1428–1441. 10.1037/pas0000623 31120298

[B40] Speight S. L. Vera E. M. (2005). University counseling center clients’ expressed preferences for counselors. *J. College Stud. Psychother.* 19 55–68. 10.1300/J035v19n03_06

[B41] Spielberger C. D. Gorsuch R. L. Lushene R. Vagg P. R. Jacobs G. A. (1983). *Manual for the State-Trait Anxiety Inventory.* Palo Alto, CA: Consulting Psychologists Press.

[B42] Steffanowski A. Oppl M. Meyerberg J. Schmidt J. Nübling R. (2001). “Psychometrische Überprüfung einer deutschsprachigen Version des Relationship Scales Questionaire (RSQ),” in *Störungsspezifische Therapieansätze—Konzepte und Ergebnisse*, ed. Bassler M. (Germany: Psychosozial), 320–342.

[B43] Swift J. K. Greenberg R. P. (2012). Premature discontinuation in adult psychotherapy: A meta-analysis. *J. Consult. Clin. Psychol.* 80 547–559. 10.1037/a0028226 22506792

[B44] Swift J. K. Callahan J. L. Vollmer B. M. (2011). Preferences. *J. Clin. Psychol.* 67 155–165. 10.1002/jclp.20759 21120917

[B45] Swift J. K. Callahan J. L. Cooper M. Parkin S. R. (2018). The impact of accommodating client preference in psychotherapy: A meta-analysis. *J. Clin. Psychol.* 74 1924–1937. 10.1002/jclp.22680 30091140

[B46] Swift J. K. Callahan J. L. Tompkins K. A. Connor D. R. Dunn R. (2015). A delay-discounting measure of preference for racial/ethnic matching in psychotherapy. *Psychotherapy* 52 315–320. 10.1037/pst0000019 26301422

[B47] Volders A. (2021). *Déterminer les préférences thérapeutiques d’un⋅e patient⋅e: Validation de la version française de l’Inventaire des Préférences de Cooper-Norcross [Liège Université].* Liège: Université de Liège.

[B48] Wild D. Grove A. Martin M. Eremenco S. McElroy S. Verjee-Lorenz A. (2005). Principles of good practice for the translation and cultural adaptation process for patient-reported outcomes (PRO) measures: Report of the ISPOR Task Force for Translation and Cultural Adaptation. *Value Health* 8 94–104. 10.1111/j.1524-4733.2005.04054.x 15804318

[B49] Williams R. Farquharson L. Palmer L. Bassett P. Clarke J. Clark D. M. (2016). Patient preference in psychological treatment and associations with self-reported outcome: National cross-sectional survey in England and Wales. *BMC Psychiatry* 16:4. 10.1186/s12888-015-0702-8 26768890 PMC4714467

[B50] Zortea T. C. Gray C. M. O’Connor R. C. (2019). Adult attachment: Investigating the factor structure of the Relationship Scales Questionnaire. *J. Clin. Psychol.* 75 2169–2187. 10.1002/jclp.22838 31332813

