# Corrigendum: Assessing Patient Preferences: Examination of the German Cooper-Norcross Inventory of Preferences

**DOI:** 10.3389/fpsyg.2022.854955

**Published:** 2022-02-22

**Authors:** Peter Eric Heinze, Florian Weck, Franziska Kühne

**Affiliations:** Clinical Psychology and Psychotherapy, University of Potsdam, Potsdam, Germany

**Keywords:** psychotherapy, preference, activity preference, preference assessment, validation study

In the original article the Funding statement was not included. The correct Funding statement is included below:

## Funding

This work received funding from Deutsche Forschungsgemeinschaft (DFG, German Research Foundation, Open Access Publication Fund), no. 491466077 to PH.

In the original article there was also an error in *Materials and Methods, Data Analyses, Paragraph 3, Footnote 1*. The preliminary URL was provided. The correct URL is “https://osf.io/n6xbq”.

The authors apologize for this error and state that this does not change the scientific conclusions of the article in any way. The original article has been updated.

## Publisher's Note

All claims expressed in this article are solely those of the authors and do not necessarily represent those of their affiliated organizations, or those of the publisher, the editors and the reviewers. Any product that may be evaluated in this article, or claim that may be made by its manufacturer, is not guaranteed or endorsed by the publisher.

