# Supplementary material for: Assessing Patient Preferences: Examination of the German Cooper-Norcross Inventory of Preferences
Source: Front Psychol. 2022 Jan 14;12:795776. doi: 10.3389/fpsyg.2021.795776 (PMC8795584; doi:10.3389/fpsyg.2021.795776)
Supplement: Supplementary file 1 [file Data_Sheet_1.docx]

**Supplementary Material**

**Supplement 1. Cooper – Norcross Inventar für Präferenzen (C-NIP)**

Bitte geben Sie bei jeder der folgenden Fragen Ihre Präferenz dafür an, wie ein/e Psychotherapeut/in mit Ihnen arbeiten sollte, indem Sie die zutreffende Zahl ankreuzen. Eine 3 entspricht einer starken Präferenz in die jeweilige Richtung, eine 2 entspricht einer moderaten Präferenz in die jeweilige Richtung, eine 1 entspricht einer leichten Präferenz in die jeweilige Richtung und eine 0 entspricht keiner Präferenz in eine Richtung bzw. eine gleich starke Präferenz in beide Richtungen.

**„Ich würde mir wünschen, der/die Therapeut/in…“**

| 1. | konzentriert sich auf spezifische Ziele | | | | keine oder gleiche Präferenz | | | | | konzentriert sich nicht auf spezifische Ziele | | | |
| --- | --- | --- | --- | --- | --- | --- | --- | --- | --- | --- | --- | --- | --- |
|  |  | 3 | 2 | 1 | | | | 0 | -1 | -2 | -3 | |  |
|  |  |  |  |  | | | |  |  |  |  | |  |
| 2. | strukturiert die Therapie | | | | keine oder gleiche Präferenz | | | | | lässt die Therapie unstrukturiert | | | |
|  |  | 3 | 2 | 1 | | | | 0 | -1 | -2 | -3 | |  |
|  |  |  |  |  | | | |  |  |  |  | |  |
| 3. | vermittelt mir Fertigkeiten zum Umgang mit meinen Problemen | | | | keine oder gleiche Präferenz | | | | | vermittelt mir keine Fertigkeiten zum Umgang mit meinen Problemen | | | |
|  |  | 3 | 2 | 1 | | | | 0 | -1 | -2 | -3 | |  |
|  |  |  |  |  | | | |  |  |  |  | |  |
| 4. | gibt mir „Hausaufgaben“ auf | | | | keine oder gleiche Präferenz | | | | | gibt mir keine „Hausaufgaben“ auf | | | |
|  |  | 3 | 2 | 1 | | | | 0 | -1 | -2 | -3 | |  |
|  |  |  |  |  | | | |  |  |  |  | |  |
| 5. | übernimmt die Führungsrolle in der Therapie | | | | keine oder gleiche Präferenz | | | | | ermöglicht, dass ich die Führungsrolle in der Therapie übernehme | | | |
|  |  | 3 | 2 | 1 | | | | 0 | -1 | -2 | -3 | |  |
|  |  |  |  |  | | | |  |  |  |  | |  |
| 6. | ermutigt mich, auf für mich schwierige Gefühle einzugehen | | | | keine oder gleiche Präferenz | | | | | ermutigt mich nicht, auf für mich schwierige Gefühle einzugehen | | | |
|  |  | 3 | 2 | 1 | | | | 0 | -1 | -2 | -3 | |  |
|  |  |  |  |  | | | |  |  |  |  | |  |
| 7. | spricht mit mir über die therapeutische Beziehung | | | | keine oder gleiche Präferenz | | | | | spricht nicht mit mir über die therapeutische Beziehung | | | |
|  |  | 3 | 2 | 1 | | | | 0 | -1 | -2 | -3 | |  |
|  |  |  |  |  | | | |  |  |  |  | |  |
| 8. | konzentriert sich auf unsere Beziehung | | | | keine oder gleiche Präferenz | | | | | konzentriert sich nicht auf unsere Beziehung | | | |
|  |  | 3 | 2 | 1 | | | | 0 | -1 | -2 | -3 | |  |
|  |  |  |  |  | | | |  |  |  |  | |  |
| 9. | ermutigt mich, starke Gefühle auszudrücken | | | | keine oder gleiche Präferenz | | | | | ermutigt mich nicht, starke Gefühle auszudrücken | | | |
|  |  | 3 | 2 | 1 | | | | 0 | -1 | -2 | -3 | |  |
|  |  |  |  |  | | | |  |  |  |  | |  |
| 10. | konzentriert sich hauptsächlich auf  meine Gefühle | | | | | keine oder gleiche Präferenz | | | | konzentriert sich hauptsächlich auf  meine Gedanken | | | |
|  |  | 3 | 2 | 1 | | | | 0 | -1 | -2 | -3 | |  |
|  |  |  |  |  | | | |  |  |  |  | |  |
| 11. | konzentriert sich auf mein  Leben in der Vergangenheit | | | | | | keine oder gleiche Präferenz | | | konzentriert sich auf  mein gegenwärtiges Leben | | | |
|  |  | 3 | 2 | 1 | | | | 0 | -1 | -2 | -3 | |  |
|  |  |  |  |  | | | |  |  |  |  | |  |
| 12. | hilft mir, über meine Kindheit nachzudenken | | | | | | keine oder gleiche Präferenz | | | hilft mir, über mein  Leben als Erwachsener nachzudenken. | | | |
|  |  | 3 | 2 | 1 | | | | 0 | -1 | -2 | -3 | |  |
|  |  |  |  |  | | | |  |  |  |  | |  |
| 13. | konzentriert sich auf meine Vergangenheit | | | | | | keine oder gleiche Präferenz | | | konzentriert sich auf meine Zukunft | | | |
|  |  | 3 | 2 | 1 | | | | 0 | -1 | -2 | -3 | |  |
|  |  |  |  |  | | | |  |  |  |  | |  |
| 14. | verhält sich schonend | | | | | | keine oder gleiche Präferenz | | | verhält sich fordernd | | | |
|  |  | 3 | 2 | 1 | | | | 0 | -1 | -2 | -3 | |  |
|  |  |  |  |  | | | |  |  |  |  | |  |
| 15. | ist unterstützend | | | | | | keine oder gleiche Präferenz | | | ist konfrontierend | | | |
|  |  | 3 | 2 | 1 | | | | 0 | -1 | -2 | -3 | |  |
|  |  |  |  |  | | | |  |  |  |  | |  |
| 16. | unterbricht mich nicht | | | | | | keine oder gleiche Präferenz | | | unterbricht mich und hilft mir,  mich zu fokussieren | | | |
|  |  | 3 | 2 | 1 | | | | 0 | -1 | -2 | -3 |  | |
|  |  |  |  |  | | | |  |  |  |  |  | |
| 17. | hinterfragt meine eigenen  Überzeugungen und Ansichten nicht | | | | | | keine oder gleiche Präferenz | | | hinterfragt meine eigenen  Überzeugungen und Ansichten | | | |
|  |  | 3 | 2 | 1 | | | | 0 | -1 | -2 | -3 | |  |
|  |  |  |  |  | | | |  |  |  |  | |  |
| 18. | unterstützt mein Verhalten bedingungslos | | | | | | keine oder gleiche Präferenz | | | hinterfragt mein Verhalten, wenn er/sie denkt, dass es falsch ist | | | |
|  |  | 3 | 2 | 1 | | | | 0 | -1 | -2 | -3 | |  |
|  |  |  |  |  | | | |  |  |  |  | |  |

| *Supplement 2: Factor Loadings of the Fitted ESEM-Model (alternative factor structure)* | | | | | |
| --- | --- | --- | --- | --- | --- |
| Nr. | Item | TD-CD | EI-ER | PaO-PrO | WS-FC |
| 1 | Focus on goals vs. Not focus on goals | **.71** | .00 | -.03 | .15 |
| 2 | Give structure vs. Allow unstructured | **.68** | .03 | -.06 | .04 |
| 3 | Teach skills vs. Not teach skills | **.87** | -.05 | -.02 | -.03 |
| 4 | Give homework vs. Not give homework | **.52** | .11 | -.03 | .00 |
| 5 | Take lead vs. Allow client lead | **.43** | .00 | .12 | .04 |
| 6 | Encourage difficult emotions vs. Not encourage | **.65** | .16 | .11 | -.08 |
| 7 | Talk about relationship vs. Not talk | .13 | **.73** | -.05 | .00 |
| 8 | Focus on therapy relationship vs. Not focus on therapy relationship | -.04 | **.70** | .03 | .07 |
| 9 | Encourage strong feeling vs. Not encourage | **.40** | **.32** | .15 | -.05 |
| ~~10~~ | ~~Focus on feelings vs. Focus on thoughts~~ |  |  |  |  |
| 11 | Focus on past vs. Focus on present | .04 | -.01 | **.87** | .01 |
| 12 | Reflect childhood vs. Reflect adulthood | .00 | .06 | **.84** | -.01 |
| 13 | Focus on past vs. Focus on future | -.05 | -.01 | **.90** | .02 |
| 14 | Be gentle vs. Be challenging | .03 | .00 | .21 | **.32** |
| ~~15~~ | ~~Supportive vs. Confrontational~~ |  |  |  |  |
| 16 | Not interrupt vs. Interrupt | .20 | -.01 | .22 | **.37** |
| 17 | Not challenge beliefs and views vs. Challenge beliefs and views | -.01 | -.10 | -.02 | **.76** |
| 18 | Support behavior unconditionally vs. Challenge behavior | **-**.21 | .08 | .01 | **.71** |
| *Note:* Exploratory Factor Analysis with Geomin-Rotation. Bold numbers indicate factor loadings > .30. TD-CD: Therapist vs. Client Directiveness; EI-ER = Emotional Intensity vs. Reserve; PaO-PrO = Past vs. Present Orientation; WS-FC = Warm Support vs. Focused Challenge. Horizontal lines separate the factors according to the original English version. | | | | | |
|  |  |  |  |  |  |
|  |  |  |  |  |  |

| *Supplement 3: Descriptive Statistics of and Correlations with Alternative C-NIP Scales* | | | | | | | | | | |
| --- | --- | --- | --- | --- | --- | --- | --- | --- | --- | --- |
| Scale | | *M* | *SD* | *α* |  | *r(TD-CD)* | *r(RI-RR)* | *r(PaO-PrO)* | *r(WS-FC)* |  |
| C-NIP | |  |  |  |  |  |  |  |  |  |
|  | Therapist vs. Client Directiveness | 10.53 | 6.97 | .82 |  | 1 |  |  |  |  |
|  | Relationship Intensity vs. Reserve | 1.89 | 2.65 | .67^1^ |  | **.43***** | 1 |  |  |  |
|  | Past vs. Present Orientation | -0.53 | 4.39 | .89 |  | **.19***** | **.18***** | 1 |  |  |
|  | Warm Support vs. Focused Challenge | -2.42 | 4.26 | .63 |  | **-.11***** | -.01 | **.34***** | 1 |  |
| Relationship Scales Questionnaire | |  |  |  |  |  |  |  |  |  |
|  | Anxiety | 2.43 | 0.91 | .85 |  | -.07* | -.04 | **.14***** | .09** |  |
|  | Avoidance | 2.37 | 0.86 | .77 |  | **-.11***** | -.06 | .06 | .10** |  |
| General Self-Efficacy | |  |  |  |  |  |  |  |  |  |
|  | Overall | 3.97 | 0.72 | .89 |  | .06 | .02 | -.07* | -.05 |  |
| Locus of Control | |  |  |  |  |  |  |  |  |  |
|  | Internal | 3.93 | 0.77 | .68^1^ |  | **.15***** | .03 | -.04 | -.01 |  |
|  | External | 2.35 | 0.84 | .58^1^ |  | -.09** | -.04 | .06 | **.12***** |  |
| Trait Anxiety | |  |  |  |  |  |  |  |  |  |
|  | Overall | 2.08 | 0.62 | .95 |  | **-.10**** | -.08* | .08* | .09** |  |
| Temporal Focus | |  |  |  |  |  |  |  |  |  |
|  | Past | 3.75 | 1.15 | .92 |  | -.03 | -.02 | **.15***** | .03 |  |
|  | Present | 4.92 | 1.08 | .90 |  | .05 | .08* | **-.11***** | -.05 |  |
| Big Five | |  |  |  |  |  |  |  |  |  |
|  | Extraversion | 3.33 | 0.95 | .85 |  | .06 | .09** | .02 | -.08* |  |
|  | Agreeableness | 3.16 | 0.78 | .65 |  | .04 | .08* | -.02 | -.01 |  |
|  | Conscientiousness | 3.72 | 0.74 | .73 |  | .08* | .05 | -.01 | -.01 |  |
|  | Neuroticism | 3.09 | 0.98 | .82 |  | -.08** | -.08* | .08* | .09** |  |
|  | Openness | 4.05 | 0.69 | .75 |  | .02 | .07* | -.01 | -.07* |  |
| *Note:* Correlations show Pearson’s correlation coefficients. Negative correlations resemble increasing preference towards the right anchor of each C-NIP’s scales. Bold correlation coefficients mark (at least) small effect sizes (*r* > .10) TD-CD = Therapist vs. Client Directiveness; RI-RR = Relationship Intensity vs. Reserve; PaO-PrO = Past vs. Present Orientation; WS-FC = Warm Support vs. Focused Challenge.  ^1^ Corrected Spearman-Brown coefficient due to 2 items per factor.  * *p* < .05. ** *p* < .01. *** *p* < .001. | | | | | | | | | | |
